# Real-time changes in player-perceived psychological momentum across scoring runs in volleyball: a single-match case study

**DOI:** 10.3389/fspor.2026.1903257

**Published:** 2026-09-16

**Authors:** Yusuke Asai, Tomoyasu Okuda

**Affiliations:** 1 Department of Sport Cultural Studies, Hokkaido University of Education, Iwamizawa-shi, Hokkaido, Japan; 2 Graduate School of Science and Technology, Kyoto Institute of Technology, Kyoto, Japan

**Keywords:** *in situ* measurement, match outcome, *Nagare*, performance dynamic, scoring streaks

## Abstract

**Introduction:**

Psychological momentum (PM) has been proposed to be associated with athletic performance, yet athletes’ real-time experiences of PM during competition remain understudied. This single-match case study examined changes in male high school volleyball players’ perceived PM across scoring runs and whether these patterns differed between two teams.

**Methods:**

Seventeen players (Team A, *n* = 8; Team B, *n* = 9) participated in a research-organized intra-squad match. After each rally, players verbally rated their own team's PM on a 7-point scale. Immediate within-player PM changes following the first, second, and third points of scoring runs were analyzed using a cross-classified linear mixed-effects model with participant and team-by-rally event as random effects. An AR(1) sensitivity analysis and an exploratory analysis of cumulative PM change were also conducted.

**Results:**

In the primary analysis, Team, Run position, and their interaction were not significant, and no between-team differences were observed at any run position. Thus, the hypothesis that the eventual winning team would show greater PM increases was not supported. Immediate PM changes were descriptively positive following own-team scoring. The AR(1) sensitivity analysis showed a significant Run-position effect, indicating model-dependent inference. Exploratorily, cumulative PM change increased across scoring-run positions, with no Team or interaction effects.

**Conclusion:**

In this single-match case, PM changes were not clearly differentiated by eventual match outcome. PM may instead reflect proximal scoring events and accumulate across successive successes, although these findings should be interpreted cautiously given the limited number of independent scoring events.

## 1 Introduction

Several scholars have proposed that psychological momentum (PM) influences athletic performance (1, 2); however, findings remain inconclusive (3). Although momentum has been broadly discussed in sport, the term encompasses several distinct research perspectives. First, psychological momentum refers to a psychological force or state arising in athletes in response to preceding successes, failures, or other events, with potential consequences for subsequent cognition, effort, behavior, and performance (1, 2). Second, strategic momentum refers to momentum arising when objective features of the competitive structure, such as accumulated competitive outcomes, the current competitive position, and remaining opportunities, alter subsequent strategic incentives and behavior (6). Third, behavioral momentum, which originates from behavioral analysis, concerns the extent to which previously reinforced behavior persists in the face of disruption or adversity—that is, resistance to behavioral change—and has also been examined in sport settings such as basketball (5). Although these perspectives share the term momentum, they differ in their theoretical foundations, units of analysis, and methods of measurement. The present study focuses specifically on psychological momentum as experienced by athletes themselves. Accordingly, when the present article refers to inconsistent findings concerning the relationship between PM and athletic performance (3), this refers primarily to the psychological momentum literature rather than to momentum research as a whole.

Research has predominantly focused on spectator perceptions of momentum based on the premise that it impacts performance (4, 7, 8). Although differences in cognitive strategies and competitive experiences have been considered (1, 7, 8), empirical support for these hypotheses remains limited. One challenge in PM research therefore concerns whose perception is being measured. Observer-based assessments are useful for examining relationships between scoring sequences, competitive situations, and perceived PM; however, they do not directly capture how athletes actually experience PM while participating in competition. Athletes compete while simultaneously experiencing not only the score but also their own and their teammates’ performances, interactions with opponents, and the preceding course of the match. This reliance on observer judgment necessitates a shift toward examining players’ direct perceptions during play to achieve a more accurate understanding of momentum. As players actively engage in competitive contexts, their subjective experiences offer critical insights into how momentum evolves during matches.

In considering the development of PM, attention should be given not only to a single success but also to successes occurring repeatedly within a short period of time. Theoretical accounts of PM have regarded preceding success as an important trigger for the emergence or change of PM (1, 2). Volleyball is particularly suited to examining temporally successive success events because each rally produces a clearly identifiable outcome in the form of a point won or conceded. In particular, when one point is followed by a second and then a third consecutive point by the same team, successful events are repeated within a short period of time. Examining how players’ perceived PM changes across the first, second, and third points of a scoring run may therefore contribute to understanding how PM develops and changes during match play. However, few studies have measured PM rally by rally among athletes actively participating in competition and examined these perceptions in relation to such scoring sequences.

Furthermore, the relationship between PM perceived during a match and the eventual match outcome remains unclear. If PM is associated with success during competition, it is conceivable that the team that ultimately wins the match may experience more positive PM during play than the team that ultimately loses. At the same time, the final match outcome results from the accumulation of numerous scoring events, and any difference observed between two teams in a single match cannot be generalized as a difference between winning and losing teams in general. Accordingly, a case-study approach involving a single closely contested match, in which players’ own perceptions of PM are assessed immediately after each rally and examined in relation to scoring sequences, may provide a useful means of characterizing these dynamics in detail.

Thus, in the present case study aimed to assess male high school volleyball players’ perceptions of their own team's PM in real time immediately after each rally and to examine changes in PM associated with the first, second, and third points of scoring runs. We further examined whether these patterns of PM change differed between Team A, which ultimately won the closely contested match, and Team B, which ultimately lost. As a working hypothesis, we expected Team A to exhibit more positive PM changes than Team B.

## 2 Methods

### 2.1 Participants and match setting

The participants included 18 male high school volleyball players belonging to the same school team. For the present study, the head coach divided the players into two intrasquad teams with the intention of balancing overall playing ability between the teams. The match was organized specifically for the purposes of this study and was not part of an official competition or league. Each team fielded seven players, including a libero, with available substitutes as necessary.

One participant was excluded because no analyzable voice-recorded PM data were obtained. Although the participant believed that the recorder had been activated, the responses had not actually been recorded. Thus, exclusion was necessary because no PM ratings were available for analysis from this participant. Data from the remaining 17 players were analyzed (Team A, *n* = 8; Team B, *n* = 9).

### 2.2 Measurement of in-match momentum

Each player individually assessed his perception of his team's momentum during play. Each participant recorded momentum ratings immediately following each rally using a 7-point Likert scale (1 = no momentum at all for my team, 4 = no difference in momentum between teams, and 7 = a lot of momentum for my team) via an IC recorder taped to the upper arm. During data collection, the Japanese term *Nagare* (流れ) was used as the operational label for PM and was defined as the perceived superiority or inferiority of a match's progression (4).

Before the match, the first author provided the participants with brief instructions regarding the operation of the IC recorder. The timing of the ratings and the use of the 7-point momentum scale were also explained before data collection. Despite these instructions, one participant believed that the recorder had been activated although no responses were actually recorded, as described in Section 2.1.

Among the 17 players included in the analysis, 476 post-rally ratings were expected across the 28 rallies (Team A, 224; Team B, 252). Of these, 444 ratings were available for analysis (Team A, 211; Team B, 233), corresponding to an overall completion rate of 93.3%. Thirty-two post-rally ratings were unavailable because corresponding voice recordings were not available (Team A, 13; Team B, 19). Missing ratings were not imputed. For analyses of immediate within-player PM change, a change score was calculated only when ratings were available for both the current rally and the immediately preceding rally.

The match was video-recorded to verify match events and score progression.

During the match, Team B scored five consecutive points in the early stages, followed by a three-point run by Team A. Subsequently, a back-and-forth scoring sequence ensued. Midway through the match, Team A achieved another three-point run, leveling the score at 9–9. The match continued with alternating points before Team A ultimately secured a 15–13 victory (Figures 1, 2). This methodological approach was designed and executed in accordance with the procedures outlined by Asai et al. (9). Although the real-time voice-recording procedure was adapted from Asai et al. (9), the present case study analyzes a different dataset and addresses a distinct research question, namely how players’ real-time PM changes vary across scoring-run positions and whether these patterns differ between the two teams in a single closely contested match.

**Figure 1:**
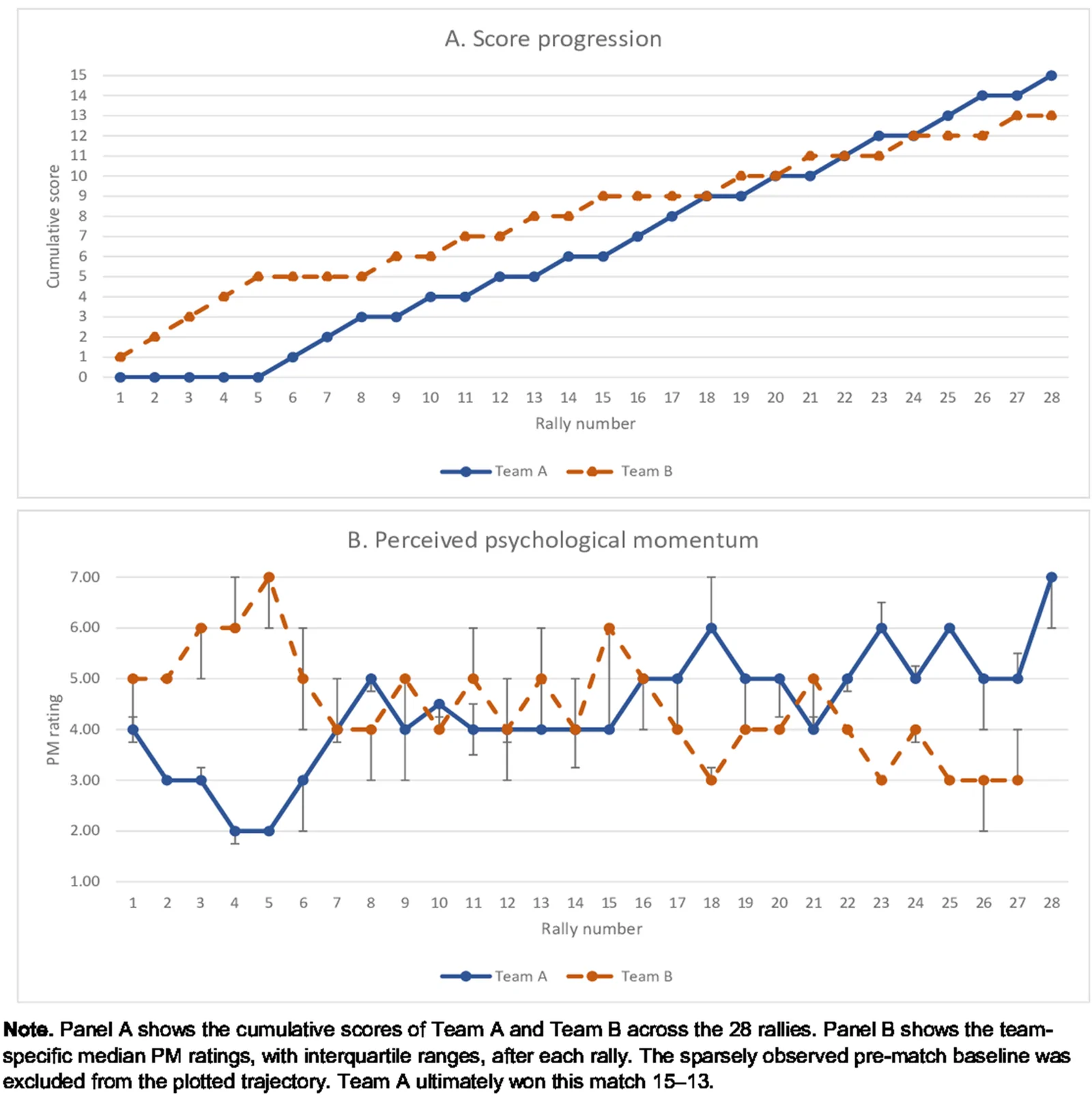
Rally-by-rally score progression and perceived psychological momentum during the single match.

**Figure 2:**
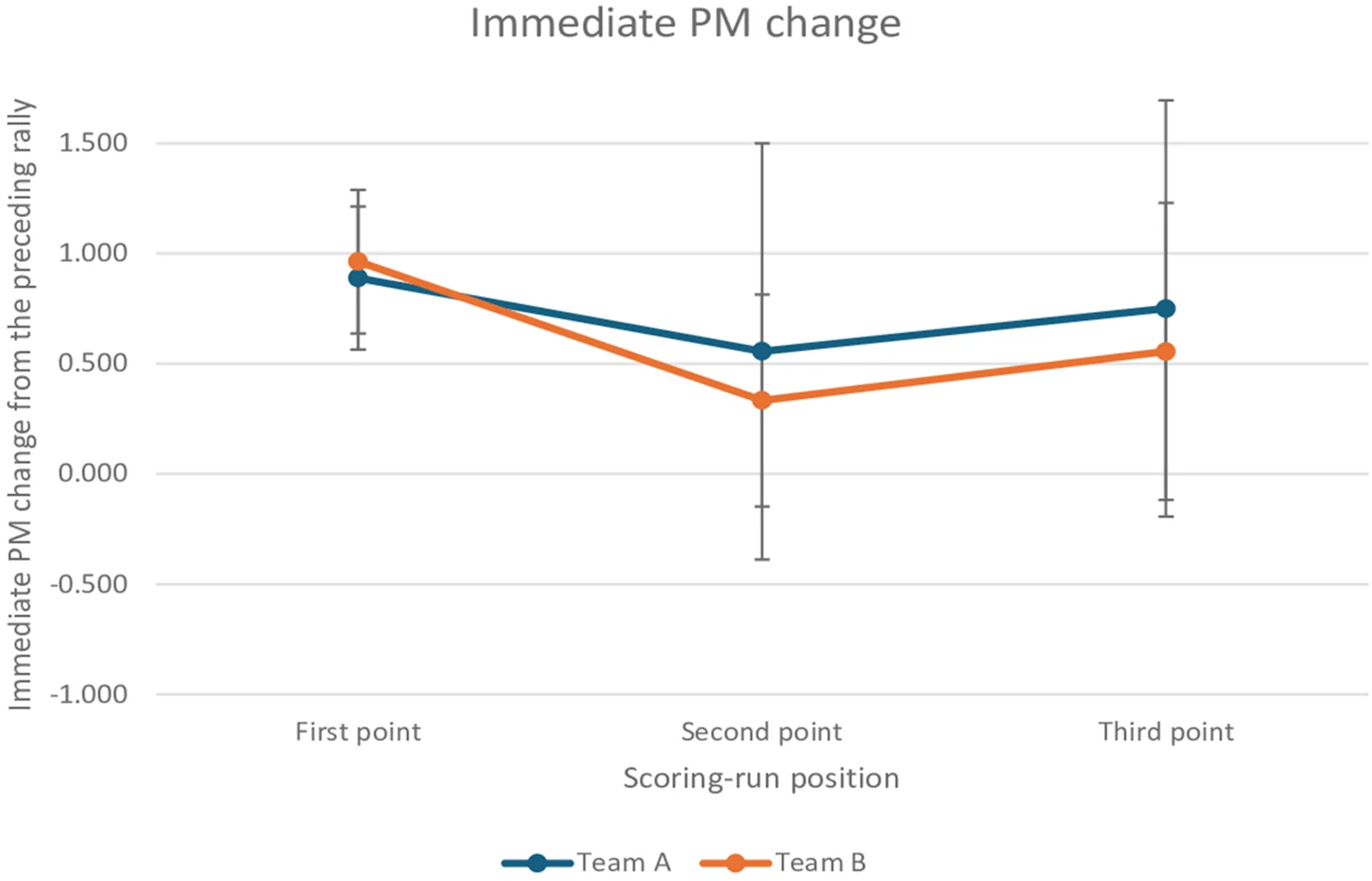
Model-adjusted immediate within-player changes in perceived psychological momentum across scoring-run positions.

### 2.3 Statistical analysis

#### 2.3.1 Data preparation and descriptive analysis

For descriptive purposes, rally-by-rally perceived psychological momentum (PM) was summarized separately for each team using the median and interquartile range (IQR). The sparsely observed pre-match (0–0) baseline was excluded from the descriptive trajectory, and the 28 post-rally observations were used to describe changes in match score and perceived PM over the course of the match.

For the inferential analyses, the data were organized at the player-by-rally level. Immediate within-player PM change was calculated as the participant's PM rating after the current rally minus that same participant's rating after the immediately preceding rally. Thus, positive values indicated an increase in perceived PM and negative values indicated a decrease. No missing ratings were imputed, and an immediate change score was calculated only when PM ratings were available for both the current and immediately preceding rallies.

A scoring run was defined as an uninterrupted sequence of points scored by the same team. For each own-team scoring event, run position was classified as the first, second, or third point of the scoring run. Points beyond the third position were not included in the primary analysis. Player-event observations represent repeated measurements and were therefore not treated as independent participants. After applying these event-selection criteria, 192 player-event observations were included in the primary analysis.

#### 2.3.2 Primary analysis of immediate PM change

The primary analysis examined whether immediate within-player PM change differed according to team, scoring-run position, or their interaction. A cross-classified linear mixed-effects model was fitted with immediate PM change as the dependent variable. Team (Team A vs. Team B), scoring-run position (first, second, or third point), and the Team × Run position interaction were specified as fixed effects.

Because each participant contributed repeated PM ratings and multiple participants from the same team responded to the same scoring event, random intercepts were specified for both participant and team-by-rally event. The latter random effect accounted for the shared context among players who experienced the same team-specific scoring event. These two clustering factors were crossed rather than nested.

Because the study comprised a single match involving only two teams, Team was treated as a case-specific fixed factor. Accordingly, the Team effect represents a comparison between Team A and Team B in this match and was not interpreted as a population-level comparison between winning and losing teams.

Models were estimated using restricted maximum likelihood (REML), and denominator degrees of freedom were estimated using the Kenward–Roger method. Fixed effects were evaluated using Type III *F* tests. Estimated marginal means, standard errors, and 95% confidence intervals were calculated for each Team × Run position combination. Team contrasts at each scoring-run position were also estimated, with Sidak adjustment for pairwise comparisons. All tests were two-sided, with statistical significance set at *p* < .05.

#### 2.3.3 Sensitivity and exploratory analyses

To examine whether the findings were sensitive to temporal dependence among repeated observations, a sensitivity model was fitted for the same immediate PM-change outcome. This model retained Team, scoring-run position, and their interaction as fixed effects and included a participant random intercept together with a first-order autoregressive [AR(1)] residual covariance structure across rallies within participants. The team-by-rally event random intercept used in the primary cross-classified model was not included in this sensitivity model. Given the limited independent event-level information available in this single-match dataset, particularly at later scoring-run positions, the shared team-by-rally event random effect and the AR(1) residual structure were not jointly estimated within the same model. Instead, temporal dependence was examined in a separate sensitivity model to limit model complexity relative to the available event-level information and to assess whether fixed-effect inferences were sensitive to an alternative plausible specification of the dependence structure.

An additional exploratory analysis examined cumulative within-player PM change across scoring runs. Cumulative change was defined as the participant's PM rating at each scoring-run position minus that participant's PM rating immediately before the scoring run began. Only observations for which the pre-run baseline was available were included. The model included Team, scoring-run position, and their interaction as fixed effects, a participant random intercept, and an AR(1) residual covariance structure within each participant-by-scoring-run sequence. As in the primary analysis, models were estimated using REML with Kenward–Roger degrees of freedom, and estimated marginal means and 95% confidence intervals were obtained.

All statistical analyses were conducted using IBM SPSS Statistics, version 29.

### 2.4 Ethical considerations

This study was approved by the Ethics Review Committee of Hokkaido University of Education (Approval No. 2022065001). Before obtaining informed consent, the players and coaches were fully informed about the study objectives, procedures, personal information protection, and voluntary participation.

## 3 Results

### 3.1 Match characteristics and data availability

Figure 1 shows the rally-by-rally score progression and perceived PM ratings for both teams across the single match. Team B led during the early stages of the match, whereas Team A subsequently reduced the deficit and eventually won the match 15–13 after 28 rallies.

Table 1 summarizes participant-level data availability and exposure to scoring-run positions. Among the 17 players included in the analysis, 476 post-rally PM ratings were expected across the 28 rallies, of which 444 were available for analysis (93.3%). A total of 415 immediate within-player PM changes could be calculated from pairs of consecutive available ratings. Of these 415 change scores, 192 player-event observations met the primary-analysis criterion of an own-team scoring event occurring at the first, second, or third position of a scoring run (Team A, *n* = 107; Team B, *n* = 85).

**Table 1:** Participant-level data availability and scoring-run exposure in the single match.

Panel A. Participant and rating availability						
Team	Players	Expected post-rally ratings	Valid ratings	Missing ratings	Completion rate, %	Immediate shifts available
Team A	8	224	211	13	94.2	196
Team B	9	252	233	19	92.5	219
Total	17	476	444	32	93.3	415

**Panel B. Scoring-run exposure for the primary analysis**				
**Team**	**Scoring-run position**	**Players contributing**	**Unique scoring events**	**Player-event observations**
Team A	First point	8	9	61
	Second point	8	4	30
	Third point	8	2	16
Team B	First point	9	9	67
	Second point	9	1	9
	Third point	9	1	9

Expected post-rally ratings were calculated across the 28 rallies; the sparsely observed pre-match baseline was excluded. All 32 missing post-rally ratings were due to unavailable voice recordings. Player-event observations represent repeated measurements and should not be interpreted as independent participants. Unique scoring events refer to distinct team-by-rally occasions at each scoring-run position. Panel B reports exposure for the primary immediate-change analysis. The 415 immediate shifts reported in Panel A represent all calculable rally-to-rally within-player PM changes across the match, whereas the 192 player-event observations reported in Panel B represent the subset corresponding to own-team scoring events at the first, second, or third position of a scoring run. For the exploratory cumulative-change analysis, Team B estimates at the second and third scoring-run positions were based on four players from one scoring run.

For the primary analysis of own-team scoring runs, Team A contributed nine unique first-point events, four second-point events, and two third-point events, corresponding to 61, 30, and 16 player-event observations, respectively. Team B contributed nine unique first-point events and one unique event each at the second and third positions, corresponding to 67, 9, and 9 player-event observations, respectively.

### 3.2 Immediate PM change during scoring runs

The primary cross-classified linear mixed-effects model examined immediate within-player PM change following the first, second, and third points of an own-team scoring run (Table 2; Figure 2). There was no significant main effect of Team, *F*(1, 17.82) = 0.19, *p* = .666, no significant main effect of Run position, *F*(2, 17.16) = 1.78, *p* = .198, and no significant Team × Run position interaction, *F*(2, 17.16) = 0.22, *p* = .804.

**Table 2:** Cross-classified linear mixed-effects model of immediate within-player PM change following the first, second, and third points of an own-team scoring run.

Panel A. Type III tests of fixed effects				
Effect	Numerator df	Denominator df	F	*p*
Team	1	17.82	0.19	0.666
Run position	2	17.16	1.78	0.198
Team × Run position	2	17.16	0.22	0.804

**Panel B. Estimated marginal means by team and scoring-run position**						
**Team**	**Scoring-run position**	**Estimate**	**SE**	**95% CI**	**Unique scoring events**	**Player-event observations**
Team A	First point	0.888	0.156	[0.562, 1.215]	9	61
	Second point	0.556	0.229	[0.076, 1.037]	4	30
	Third point	0.750	0.321	[0.076, 1.424]	2	16
Team B	First point	0.963	0.155	[0.639, 1.287]	9	67
	Second point	0.333	0.447	[−0.610, 1.277]	1	9
	Third point	0.556	0.447	[−0.388, 1.499]	1	9

**Panel C. Team contrasts at each scoring-run position**					
**Scoring-run position**	**Team A−Team B**	**SE**	**df**	**95% CI**	***p***
First point	−0.074	0.220	19.54	[−0.534, 0.385]	0.739
Second point	0.223	0.502	17.21	[−0.835, 1.282]	0.662
Third point	0.194	0.550	17.18	[−0.965, 1.354]	0.728

The outcome was immediate within-player PM change from the preceding rally. Participant and team-by-rally event were included as crossed random intercepts. Team was treated as a case-specific fixed factor and does not represent a population-level comparison between winning and losing teams. Degrees of freedom were estimated using the Kenward–Roger method. The estimated random-intercept variances were 0.006 for participant and 0.159 for team-by-rally event; the corresponding adjusted ICCs were .012 and .305, respectively. Player-event observations represent repeated measurements and should not be interpreted as independent observations.

The estimated marginal means for Team A were 0.888 [95% CI (0.562, 1.215)] after the first point, 0.556 [95% CI (0.076, 1.037)] after the second point, and 0.750 [95% CI (0.076, 1.424)] after the third point. The corresponding estimates for Team B were 0.963 [95% CI (0.639, 1.287)], 0.333 [95% CI (−0.610, 1.277)], and 0.556 [95% CI (−0.388, 1.499)], respectively.

Direct contrasts between Team A and Team B were not significant at the first (*p* = .739), second (*p* = .662), or third (*p* = .728) scoring-run position. Thus, the primary model did not provide evidence that immediate PM changes differed between the two teams or systematically varied across the first three positions of a scoring run.

### 3.3 Sensitivity and exploratory analyses

In the sensitivity analysis incorporating an AR(1) residual covariance structure, the main effect of Run position was significant, *F*(2, 143.09) = 6.00, *p* = .003, whereas neither the main effect of Team, *F*(1, 30.06) = 1.42, *p* = .242, nor the Team × Run position interaction, *F*(2, 143.09) = 1.14, *p* = .321, was significant (Supplementary Table S1). The estimated AR(1) correlation was −0.236 [95% CI (−0.421, −0.032), *p* = .019]. Team contrasts were not significant at any scoring-run position.

The exploratory analysis of cumulative within-player PM change from the pre-run baseline showed a significant main effect of Run position, *F*(2, 77.21) = 21.06, *p* < .001 (Supplementary Table S2; Supplementary Figure S1). In contrast, neither the main effect of Team, *F*(1, 66.65) = 0.61, *p* = .437, nor the Team × Run position interaction, *F*(2, 77.21) = 1.01, *p* = .370, was significant. Estimated cumulative PM change increased across the three scoring-run positions from 0.879 to 2.431 for Team A and from 0.951 to 1.944 for Team B. No significant differences between Team A and Team B were observed at any scoring-run position. For Team B, however, estimates at the second and third positions were based on four players with complete pre-run baseline data from a single scoring run and should therefore be interpreted cautiously.

## 4 Discussion

### 4.1 PM changes during scoring runs and eventual match outcome

In the present study, players’ perceptions of their own team's PM were assessed immediately after each rally, and immediate changes in PM following the first, second, and third points of scoring runs were examined. We further investigated whether these patterns differed between Team A, which ultimately won the match, and Team B, which ultimately lost. We initially expected Team A to perceive more positive PM than Team B. However, in the primary analysis, which accounted for repeated observations within players and the fact that multiple players shared the same scoring events, neither the main effect of Team, the main effect of Run position, nor the Team × Run position interaction was significant. Thus, in this case, the original hypothesis that immediate PM changes during scoring runs would differ according to the eventual match outcome was not supported.

However, these findings should not be interpreted as indicating that PM is unrelated to match outcome. The present study did not examine the general relationship between PM and winning or losing; rather, it addressed the more specific question of whether immediate PM changes during scoring runs differed between Team A, which ultimately won this single match, and Team B, which ultimately lost. Indeed, the point estimates of PM change were positive for both teams at each run position, indicating a descriptive pattern in which perceived PM tended to shift in a positive direction following an own-team point. Nevertheless, there was no clear evidence that the magnitude of these changes differed between the two teams.

This pattern raises the possibility that real-time fluctuations in PM may be more closely related to proximal match events experienced by players at a given moment—such as scoring, score context, the preceding scoring sequence, or the content of the play leading to the point—than to the relatively distal outcome of whether the match is ultimately won or lost. Asai et al. (9) similarly reported that PM assessed during actual match play changed in association with specific successful and unsuccessful events and the content of play. Accordingly, understanding the dynamics of PM during competition may require consideration not only of the binary distinction between eventual victory and defeat, but also of the immediate match context and performance events experienced by players.

### 4.2 The first point of a scoring run and immediate PM change

Although the primary analysis did not identify a significant main effect of Run position, descriptively, both teams showed a larger immediate PM change following the first point of a scoring run than following the second or third point. This pattern suggests that scoring runs may need to be considered not simply as sequences in which the number of successive successful events increases from one to two to three, but also in terms of the distinct meaning of the first point as an event that changes the direction of the scoring sequence. In volleyball, the first point of a scoring run often terminates an opponent's preceding scoring sequence and reverses the direction of scoring toward one's own team. Thus, compared with the second or third point, which extends an already established scoring run, the first point that stops the opponent's scoring and creates the opportunity for one's own team to initiate a scoring run may represent a particularly psychologically salient event for players.

However, this interpretation was not directly supported by the primary inferential analysis and should therefore be regarded as a hypothesis generated from the descriptive pattern observed in the present case. Moreover, the nature of the immediately preceding event differs between the first and later points of a scoring run. The first point typically represents a transition from an opponent's point to an own-team point, whereas the second and third points represent transitions from an own-team point to another own-team point. The present data therefore cannot determine whether the numerically larger PM change at the first point reflects the psychological significance of reversing the scoring sequence itself or differences in the psychological state immediately preceding that point.

The use of a 7-point PM rating scale should also be considered when interpreting these changes. Because immediate PM change was calculated relative to the rating following the preceding rally, the amount of possible positive change is mechanically constrained as the preceding PM rating approaches the upper bound of the scale. For example, if the first point of a scoring run already raises a player's PM rating to a relatively high level, a subsequent point may be experienced as producing an additional increase in PM while allowing only a small numerical increase on the rating scale. This constraint inherent in a bounded scale may therefore have compressed the magnitude of observed immediate changes at the second and third points. Nevertheless, the present results cannot be attributed definitively to this measurement constraint. Future research should therefore consider measurement approaches that retain the practical advantages of rapid real-time assessment while providing greater sensitivity to acute fluctuations in PM.

In addition, the sensitivity analysis incorporating an AR(1) structure to account for temporal dependence yielded a significant main effect of Run position, in contrast to the primary analysis (Supplementary Table S1). Thus, evidence for a run-position effect was not robust across alternative model specifications. This indicates that inference regarding the association between scoring-run position and immediate PM change was sensitive to how dependence among observations was modelled. Accordingly, the present findings do not provide sufficient evidence to conclude that stable differences exist among the first, second, and third points of scoring runs. The effect of Run position should be re-examined in future studies using a larger number of independent scoring events.

### 4.3 Cumulative PM change and sampling of scoring events

In the exploratory analysis (Supplementary Table S2 and Supplementary Figure S1), cumulative PM change relative to the PM rating immediately before the scoring run showed a significant main effect of Run position. Specifically, the difference in PM from the pre-run baseline increased as the scoring sequence progressed from the first to the second and third points. In contrast, the primary analysis of immediate PM change did not provide clear evidence that each successive point produced a progressively larger point-to-point change in PM. The exploratory finding therefore should not be interpreted as indicating that the second or third point exerted a stronger psychological effect than the first point. Rather, it may indicate that changes associated with successive successful events accumulate over the course of a scoring run, producing an increasing difference from the PM level that preceded the run. The distinction between immediate point-to-point change and cumulative change across a scoring sequence emerged from the exploratory findings of the present study and should be examined as an *a priori* hypothesis in future research.

At the same time, the number of independent scoring events differed substantially across scoring-run positions. In particular, Team B contributed only one unique scoring event at each of the second and third run positions. This scarcity of events does not itself explain why the estimates for Team B were numerically lower at these positions; rather, it means that these estimates depended heavily on a single scoring sequence and were therefore inherently unstable. Consequently, the numerical differences between Team A and Team B at the second and third points should not be attributed to eventual match outcome or to team-specific characteristics of PM.

This issue also has implications for the sampling of PM in interactive sports. Simply increasing the number of participating players or player-event observations does not necessarily provide a corresponding increase in independent information about PM. Because multiple players share the same scoring event, adequate numbers of independent scoring events or scoring runs are required for each condition of interest. Conversely, simply accumulating a large number of matches may introduce a different problem. Teams with greater competitive ability are likely to generate more points and scoring runs and to maintain advantageous match states for longer periods. Consequently, high PM ratings may also be maintained for longer periods, making it increasingly difficult to distinguish acute psychological fluctuations in PM from more persistent states associated with competitive superiority.

Future PM research therefore requires more than simply increasing sample size. Sampling and modelling strategies are needed that provide adequate coverage of independent scoring events while simultaneously accounting for competitive heterogeneity, including team strength, opponent characteristics, score context, and match-level superiority. For example, studies involving multiple matches could explicitly model differences in team and match strength while separating each player's or match's typical PM level from acute deviations associated with particular scoring events. Such an event-centered research design may be important for conceptualizing PM not merely as a state in which a competitively superior team reports higher ratings, but as a dynamic psychological process that changes in relation to the evolving context of the match.

This issue is also relevant to the Japanese concept of *Nagare* (“flow of the game”). *Nagare* has been suggested to be perceived not only in relation to individual points or successful events, but also in relation to broader contextual features such as the preceding match progression, score context, and the content of play. The present study did not examine cultural differences and therefore cannot establish distinctions between psychological momentum and *Nagare*, or between Eastern and Western modes of cognition. Nevertheless, understanding acute changes in PM may require attention not only to isolated performance outcomes but also to the meaning of those events within the broader context of the match. This represents an important direction for future research.

## 5 Limitations

This study has several limitations. First, this was a single-match case study involving male high school volleyball players from the same school, with the intra-squad match organized specifically for research purposes. Therefore, the findings cannot be directly generalized to adult athletes, elite players, teams at different competitive levels, or PM experienced in official competition. In particular, an official competitive match and an intra-squad match arranged for research purposes may differ in the psychological meaning attached to winning and losing, competitive pressure, emotional involvement, and coaching intervention, all of which may influence perceived PM.

Second, the number of independent scoring events differed substantially across scoring-run positions. In particular, Team B contributed only one unique scoring event at each of the second and third scoring-run positions. Consequently, the estimates at these positions depended heavily on a limited number of scoring sequences and were associated with considerable uncertainty. This does not imply that the estimates were systematically biased in a particular direction; rather, there was insufficient independent event-level information to estimate differences between teams or run positions with high stability. Moreover, the sensitivity analysis incorporating an AR(1) structure yielded a significant effect of Run position, whereas the corresponding effect was not significant in the primary analysis. Thus, evidence for the Run-position effect was not sufficiently robust across alternative plausible model specifications. The findings concerning Run position should therefore be interpreted cautiously.

Third, PM was assessed using a single-item 7-point scale to minimize response burden during match play and enable immediate assessment after each rally. Although this approach allowed rapid and repeated measurement during competition, a single rating may not fully capture the potentially multidimensional psychological experience of PM. Furthermore, because the scale had finite upper and lower bounds, the amount of possible positive change was mechanically constrained when the preceding PM rating was already close to the upper end of the scale. This characteristic may have compressed immediate PM changes during the later stages of scoring runs. However, it cannot be concluded that this measurement constraint was responsible for the nonsignificant findings observed in the present study.

In addition, PM was assessed using the Japanese concept of *Nagare*. Although *Nagare*, as operationally defined in the present study, is closely related to PM, it may involve a more contextual and holistic appraisal incorporating factors such as score situation, match progression, and the content of play. Caution is therefore warranted in assuming that a single-item rating of *Nagare* measures exactly the same psychological construct as psychological momentum as theorized primarily in the English-language literature.

Fourth, the *in situ* procedure itself, in which players repeatedly rated PM immediately after each rally, may have influenced their experience of the match. A major advantage of this method is that it reduces the potential for retrospective memory bias and directly captures players’ experiences at the time they occur. However, explicitly evaluating PM after each rally may also have directed players’ attention more strongly than usual toward the score, their emotional state, or the perceived *Nagare* of the match. Thus, the PM perceptions observed in the present study may partly reflect measurement reactivity in addition to naturally occurring psychological experiences.

## 6 Future perspectives

Future research should prioritize research designs that secure a sufficient number of independent scoring events rather than simply increasing the number of participants or player-event observations. As demonstrated in the present study, obtaining responses from multiple players regarding the same scoring event does not mean that those observations represent independent scoring events. It is therefore important to obtain multiple independent scoring runs for each condition of interest, including the first, second, and third points of scoring runs.

At the same time, simply accumulating a large number of matches may not fully resolve this issue. Teams with greater competitive ability are likely to generate more points and scoring runs and may maintain advantageous match states for longer periods. Consequently, persistently high PM ratings may reflect either acute psychological changes associated with particular events or more sustained competitive superiority, making these processes difficult to distinguish. Future studies therefore require sampling and modelling strategies that provide adequate independent event coverage while simultaneously accounting for competitive heterogeneity, including team strength, opponent strength, score situation, match phase, and match-level superiority.

One possible approach would be to examine multiple matches involving teams of relatively comparable competitive ability and use multilevel models to distinguish each player's typical PM level, or the PM level characteristic of a particular match, from transient PM changes associated with specific scoring events. Event-centered designs may also be useful. For example, theoretically distinct events—such as a point that reverses the direction of scoring, a point that extends an existing scoring run, or a point that levels the score—could be defined *a priori* and compared across events with similar score contexts and preceding PM levels. Such approaches may help distinguish sustained competitive superiority from acute changes in PM occurring at particular moments of a match.

Measurement methods also require further development. Real-time measurement inevitably involves a trade-off between response efficiency and measurement sensitivity. Future research should therefore seek methods that are sufficiently rapid to avoid disrupting competitive behavior while providing greater sensitivity to acute PM fluctuations than a single Likert-type rating. One realistic approach would be the change-based assessment used by Asai et al. (9), in which athletes directly report the extent to which PM has changed from the preceding rally without predefined upper and lower bounds. In addition, rather than relying exclusively on difference scores between adjacent rallies, future studies could model the current PM rating while adjusting for the preceding PM level or could apply statistical models that explicitly account for the ordinal nature of the rating scale.

Finally, although the exploratory analysis did not provide clear evidence that each successive point produced a progressively larger immediate PM change, cumulative PM change relative to the pre-run baseline increased as the scoring sequence progressed. Future studies should therefore distinguish, as an *a priori* hypothesis, between the possibility that successive successes progressively amplify the psychological impact of each individual point and the possibility that psychological changes associated with successive events accumulate over time. The descriptive pattern observed in the present study, in which the first point reversing the scoring sequence was associated with a relatively large immediate PM change, also warrants direct examination in studies with sufficient numbers of independent scoring events.

## 7 Conclusion

This study assessed male high school volleyball players’ real-time perceptions of their own team's PM immediately after each rally and examined changes in PM associated with the first, second, and third points of scoring runs. In this case, no clear differences in immediate PM changes during scoring runs were observed between Team A, which ultimately won the match, and Team B, which ultimately lost; thus, the original hypothesis was not supported. These findings indicate the limitations of explaining acute changes in PM during match play solely in terms of the eventual match outcome.

At the same time, both teams showed a descriptive pattern of positive changes in PM following their own scoring, and the exploratory analysis indicated that cumulative PM change relative to the pre-run baseline increased as the scoring sequence progressed. These findings suggest that PM may change dynamically in relation to match events experienced by players at a given moment, such as individual points and scoring sequences, rather than being determined solely by the eventual match outcome. However, because this was a single-match case study and the number of independent scoring events was particularly limited at later scoring-run positions, these findings should be interpreted cautiously; in particular, the cumulative-change findings are exploratory. Future research should secure sufficient numbers of independent scoring events and employ research designs that account for competitive ability, score context, and broader match context in order to further investigate acute changes in PM during competition.

## Author’s note

As this study was conducted in Japan, the concept of momentum needed to be translated into Japanese. In this study, psychological momentum was translated as *Nagare*. Iso-Ahola and Mobily (10) defined momentum as follows: “Psychological momentum is an added or gained psychological power which changes a person's view of himself or of others or others’ view of him and of themselves. Through modified perceptions, psychological momentum influences the individual's mental and physical effort and actual behavior and performance.”

By contrast, *Nagare*, as used in this study, is defined as “the perceived superiority or inferiority of a match's progression” (4). Furthermore, in the Japanese context, emphasis is placed on factors occurring around the periphery of performance, such as “changes in the state of the game” (4) and “execution of intended plays” (11, 12). This distinction may be explained by the framework of analytic versus holistic thought in cultural psychology (13).

Analytic thought is rooted in Aristotelian logic and characterized by decoupling the object from its context, focusing on its attributes, and avoiding formal contradictions. By contrast, holistic thought is rooted in ancient Chinese Confucianism and Taoism and characterized by an orientation toward the context or the “field” as a whole and a preference for considering relationships when explaining events. Therefore, while “psychological momentum” and “*Nagare*” may describe the same underlying phenomenon, the cognitive styles used to perceive them may differ fundamentally.

Other concepts similar to momentum include “hot hand” and “clutch play,” while non-academic synonyms include “tide of the game” and “ebb and flow.” In the Japanese context, expressions such as *Ikioi* (勢い) and *Rhythm* (リズム) are also related to *Nagare*. Each of these terms likely carries distinct nuances, and even among those studied academically, verification methods remain inconsistent. Consequently, future research is encouraged to conduct comparative studies between English academic terms, such as “momentum” and “hot hand,” and the Japanese concept of *Nagare*.

## Data Availability

The raw data supporting the conclusions of this article will be made available by the authors, without undue reservation.

## Supplementary material

SUPPLEMENTARY FIGURE S1 Model-adjusted cumulative within-player PM change from the pre-run baseline across scoring-run positions.
