# Supplementary material for: Real-time changes in player-perceived psychological momentum across scoring runs in volleyball: a single-match case study
Source: Front Sports Act Living. 2026 Sep 16;8:1903257. doi: 10.3389/fspor.2026.1903257 (PMC13624159; doi:10.3389/fspor.2026.1903257)
Supplement: Supplementary file 1 [file Table1.docx]

| **Supplementary Table S1. Sensitivity analysis of immediate within-player PM change using an AR(1) residual covariance structure** | | | | |
| --- | --- | --- | --- | --- |
| Panel A. Type III tests of fixed effects | |  |  |  |
| **Effect** | **Numerator df** | **Denominator df** | **F** | **p** |
| Team | 1 | 30.06 | 1.42 | 0.242 |
| Run position | 2 | 143.09 | 6 | 0.003 |
| Team × Run position | 2 | 143.09 | 1.14 | 0.321 |
|  |  |  |  |  |
| Panel B. Estimated marginal means | |  |  |  |
| **Team** | **Scoring-run position** | **Estimate** | **SE** | **95% CI** |
| Team A | First point | 0.891 | 0.092 | [0.704, 1.077] |
|  | Second point | 0.603 | 0.126 | [0.353, 0.853] |
|  | Third point | 0.802 | 0.171 | [0.464, 1.140] |
| Team B | First point | 0.958 | 0.089 | [0.779, 1.136] |
|  | Second point | 0.34 | 0.227 | [−0.108, 0.789] |
|  | Third point | 0.554 | 0.229 | [0.103, 1.006] |
|  |  |  |  |  |
| Panel C. Team contrasts at each scoring-run position | | |  |  |
| **Scoring-run position** | **Team A − Team B** | **SE** | **95% CI** | **p** |
| First point | −0.067 | 0.128 | [−0.325, 0.191] | 0.604 |
| Second point | 0.262 | 0.258 | [−0.248, 0.772] | 0.312 |
| Third point | 0.248 | 0.285 | [−0.314, 0.810] | 0.385 |
| **Note.** This sensitivity model included a participant random intercept and an AR(1) residual covariance  structure but did not include the team-by-rally random intercept used in the primary cross-classified model. The outcome was immediate within-player PM change from the preceding rally. Accordingly, this analysis is interpreted as sensitivity evidence rather than as the primary inferential model.The estimated AR(1) correlation was −0.236 (95% CI [−0.421, −0.032], p = .019). | | | | |

| **Supplementary Table S2. Exploratory analysis of cumulative within-player PM change from the pre-run baseline across scoring-run positions** | | | | | | | |
| --- | --- | --- | --- | --- | --- | --- | --- |
| Panel A. Type III tests of fixed effects | |  |  |  |  |  |  |
| **Effect** | **Numerator df** | **Denominator df** | **F** | **p** |  |  |  |
| Team | 1 | 66.65 | 0.61 | 0.437 |  |  |  |
| Run position | 2 | 77.21 | 21.06 | < .001 |  |  |  |
| Team × Run position | 2 | 77.21 | 1.01 | 0.370 |  |  |  |
|  |  |  |  |  |  |  |  |
| Panel B. Estimated marginal means by team and scoring-run position | | |  |  |  |  |  |
| **Team** | **Scoring-run position** | **Estimate** | **SE** | **95% CI** | **Players** | **Unique scoring events** | **Player-event observations** |
| Team A | First point | 0.879 | 0.118 | [0.632, 1.127] | 8 | 9 | 61 |
|  | Second point | 1.595 | 0.144 | [1.303, 1.886] | 8 | 4 | 29 |
|  | Third point | 2.431 | 0.185 | [2.062, 2.799] | 8 | 2 | 15 |
| Team B | First point | 0.951 | 0.112 | [0.716, 1.187] | 9 | 9 | 67 |
|  | Second point | 1.446 | 0.317 | [0.818, 2.075] | 4 | 1 | 4 |
|  | Third point | 1.944 | 0.354 | [1.245, 2.643] | 4 | 1 | 4 |
|  |  |  |  |  |  |  |  |
| Panel C. Team contrasts at each scoring-run position | | |  |  |  |  |  |
| **Scoring-run position** | **Team A − Team B** | **SE** | **df** | **95% CI** | **p** |  |  |
| First point | −0.072 | 0.161 | 18.31 | [−0.409, 0.265] | 0.661 |  |  |
| Second point | 0.148 | 0.342 | 101.4 | [−0.531, 0.827] | 0.666 |  |  |
| Third point | 0.487 | 0.393 | 152.14 | [−0.290, 1.263] | 0.217 |  |  |
| **Note.** The outcome was cumulative within-player PM change from the pre-run baseline. The model included a participant random intercept and an AR(1) residual covariance  structure within player-by-run sequences. This model did not include the team-by-rally random intercept used in the primary cross-classified analysis and is therefore interpreted as exploratory evidence. For Team B, estimates at the second and third scoring-run positions were based on four baseline-complete players from a single scoring run. Because cumulative PM change incorporates changes arising from preceding points in the run, increases across scoring-run positions should not be interpreted as evidence that later individual points exerted progressively stronger effects on PM. | | | | | | | |
